# Students Supporting Critical Care – A contention plan to prevent the decompensation of ICUs in the COVID-19 pandemic:Translating Bjorn Ibsens’ polio-lessons to modern times

**DOI:** 10.1186/s13054-020-02919-1

**Published:** 2020-05-08

**Authors:** Pedro David Wendel Garcia, Paola Massarotto, Katja Auinger, Reto Andreas Schuepbach, Stephanie Klinzing

**Affiliations:** Institute of Intensive Care Medicine, University Hospital Zurich, Zurich, Switzerland

On 27 August 1952, in Copenhagen, over 3000 patients had been diagnosed with poliomyelitis, 335 with severe progression of disease and 31 with bulbar-polio in need of respiratory support, of whom 27 died about 3 days after admission [1]. That day, Bjørn Ibsen successfully tracheotomized, relaxed, and positive-pressure ventilated a dying, quadriplegic 12-year-old girl, implementing a therapy that revolutionized polio patient management, reducing mortality from 87 to 22%. Nevertheless, the key to logistically enable the ventilation of all severely paralyzed polio patients over weeks laid in the 600 nurses supported by 250 medical students, who would actuate the bellows at the bedside [2].

As of 26 March 2020, 520,395 patients worldwide have been infected by SARS-CoV-2; of these, 19,238 are in a critical state and 23,593 have died. In Italy, within a time-frame of 35 days and after 80,589 positive COVID-19 tests, 3612 patients require critical care and mortality has risen to 10.2% (8215). The developments in China and Italy have shown the incredible challenges health care systems face with this exponentially progressing pandemic [3]. In contrast to China, the distinct separation between international, national, and regional health care systems in Europe, coupled with the rapid territorial spread of COVID-19, makes a selective deployment of personnel resources to affected regions as well as a generalized quarantine impossible [4, 5].

In light of this public health emergency, Ibsen’s ideas return to critical care: we propose the training and deployment of medical students in ICUs to support the management of COVID-19 patients. By supplementing senior nurses with specifically trained medical students, a constantly growing support structure can be established. We believe that the key to success is focused training and a narrow framework of responsibility for these students; a nursing support structure is being created, not a replacement for trained intensivists.

**Table 1:** Goals, scope, and training for medical students supporting critical care management

**Overall goal** • Care and monitoring of ICU patients including COVID-19 patients under supervision of senior ICU nurses	
**Scope of responsibility** • Monitoring and reporting on: o Clinical status (temperature, GCS, heart-frequency, blood-pressure, respiratory-frequency, arterial BGA) o Respiratory support via nasal cannula, bag mask, high flow or NIV (oxygen flow and ventilation settings) o Respiratory support of intubated patients under direct supervision • Control, maintenance, and reporting on installations and infusions (central venous lines, drains, urinary catheter, etc.) • Support in emergency situations (cardiac/respiratory arrest) • Support during patient transport and medical interventions • Care of patients o Management and nursing of the conscious patient (assistance in primary needs) o Management and nursing of the unconscious patient o Bedding and mobilization o Ensuring standard hygiene and preventive measures • General support of nurses	
**Theoretical and hands-on training focus** • Confidentiality, documentation, reporting • Infectology, self-protection, hygiene, and management of infectious patients • Catheters and ICU installations • Infusions and nutrition in the ICU • Microbiological sampling, including blood samples and arterial blood gas collection • Hemodynamics o Anatomical and physiological considerations o Non-invasive and invasive blood pressure, heart-frequency assessment o Basic vasopressor and inotropic management o Cardiac and respiratory arrest • Ventilation o Anatomical and physiological considerations o High-Flow and NIV o VAP prevention o Basic mechanical ventilation management o Ventilation via bag-valve mask • Correct mobilization and bedding-position techniques (prone position) • Basic communication, ethical aspects, patient safety, and self-care in crisis situations	

Link to full resources: http://www.intensivmedizin.usz.ch/fachwissen/Seiten/COVID-19-Epidemics.aspx

The goal here is to teach a basic understanding of COVID-19 regarding hygienic standards and characteristics of patient decompensation, as well as ICU monitoring standards, supportive hemodynamic/respiratory care, and patient management and nursing. Focus should be placed on the early recognition of patient deterioration and effective reporting to senior nurses and treating physicians (Table 1).

Herewith, we provide access to our continuously developing training resources. National and international cooperation should be encouraged from the inception of this pandemic; resource-intensive logistic plans should be jointly designed and openly shared by specialized hospitals and institutions in an effort to aid rapid implementation of global containment infrastructures.

## Data Availability

All training resources and data referred to in this manuscript can be found on our repository under the following link: http://www.intensivmedizin.usz.ch/fachwissen/Seiten/COVID-19-Epidemics.aspx¨
